# Space, place and (waiting) time: reflections on health policy and politics

**DOI:** 10.1017/S1744133117000366

**Published:** 2018-02-19

**Authors:** Sally Sheard

**Affiliations:** Department of Public Health and Policy, University of Liverpool, Liverpool, UK

## Abstract

Health systems have repeatedly addressed concerns about efficiency and equity by employing trans-national comparisons to draw out the strengths and weaknesses of specific policy initiatives. This paper demonstrates the potential for explicit historical analysis of waiting times for hospital treatment to add value to spatial comparative methodologies. Waiting times and the size of the lists of waiting patients have become key operational indicators. In the United Kingdom, as National Health Service (NHS) financial pressures intensified from the 1970s, waiting times have become a topic for regular public and political debate. Various explanations for waiting times include the following: hospital consultants manipulate NHS waiting lists to maintain their private practice; there is under-investment in the NHS; and available (and adequate) resources are being used inefficiently. Other countries have also experienced ongoing tensions between the public and private delivery of universal health care in which national and trans-national comparisons of waiting times have been regularly used. The paper discusses the development of key UK policies, and provides a limited Canadian comparative perspective, to explore wider issues, including whether ‘waiting crises’ were consciously used by policymakers, especially those brought into government to implement new economic and managerial strategies, to diminish the autonomy and authority of the medical professional in the hospital environment.

Waiting lists have been a constant presence in the British National Health Service (NHS) since its formation in 1948, and in many other general tax-funded health systems including those in Canada, Australia, New Zealand and the Nordic countries.^1^ There have been regular attempts to manage waiting lists and the times people wait, especially for hospital inpatient treatment, and ‘waiting’ has become a key operational indicator for health service performance.^2^ Explanations for the existence and size of waiting lists and length of time waiting for treatment have included the inefficient use of available resources, underinvestment in the health service and clinicians manipulating their productivity to maintain demand for private practice.

This paper offers the first historical analysis of waiting for elective care in the NHS. It uses ‘waiting’ to explore wider issues in the development of health policy and delivery of health care, especially the changing authority of medical professionals, health economists and health service management. It tests a broad hypothesis that as pressures on NHS expenditure increased, especially from the 1960s (due to general economic crises as well as rising demand for more expensive treatments by an ageing population) politicians responded by shifting the policy focus from waiting lists to waiting times, and by forming new collaborations with health economists, who benefited professionally from the increased use of their expertise. Medical professionals, in contrast, appeared to have little to bring to the policy table on waiting lists/times, or little willingness or invitation to do so.

Some policy theorists have approached the issue of performance management in health services through broad categorisation into ‘intelligence’, ‘targets’ and ‘rankings’ (Hood, 2007); others have focussed on how opportunities are created (and taken) for policy change by considering actors, their collaborations and the political climates in which they operate (Sabatier, 1988; Wilsford, 1994; Kingdon, 1995; Tuohy, 1999, 2017a, 2017b). None has adequately considered the role of relative expert status, or how to accommodate the vital issue of ‘space’ (dynamic impact of sub-national variations in performance) in evaluating NHS policy development. Three key policy lessons emerge from the history of waiting lists and times in the United Kingdom. First, a failure to acknowledge and address tensions between the different NHS policy levels (intra-/inter-regional and national) will affect the relative success of performance management techniques. Second, radical policy changes should be piloted, even if this is uncomfortable/risky for policymakers. Third, conscious effort needs to be invested by policymakers in ensuring all expert groups continue to be actively engaged in policy formation.

The following sections of this paper establish first, the trajectory of waiting lists and times in the UK NHS, and responses to them from three main groups of actors: government ministers, medical professionals and health economists. Second, it considers the points at which significant policy changes on waiting lists were proposed or attempted, and what factors influenced their relative uptake. Third, it reflects on how variations in waiting lists and times expose the ‘sub-national’ nature of the British NHS; their active role in shaping services, and what this can tell us about the validity and impact of targets and standards as governance tools. Fourth, it offers some broader comment on the logic of comparative studies in health policy − and how ‘time’ (i.e. history) can be a valuable analytic alongside ‘space’ (comparative studies) and ‘place’ (institutional studies), and provides a brief overview of the Canadian history of waiting in health care. Since waiting times are a notable feature of Beveridge-style health systems and the British NHS is the oldest of these systems, this history offers policy relevance beyond the United Kingdom.

## The NHS: 1948–1979

When the NHS began on 5 July 1948, there were widespread concerns that there would be queues outside hospitals and general practitioner (GP) surgeries of patients with chronic health care needs. Queues did not form, but there have been waiting lists ever since. The types of patients waiting has changed in line with medical developments: waiting lists for thoracoplasty for TB disappeared with the introduction of streptomycin in 1943; waiting lists for hip replacements did not appear until John Charnley developed a successful hip prosthesis in 1962. Heart attack patients who once went onto waiting lists for investigation now often go straight to theatre from the ambulance. For elective surgery, which is one of the largest items of NHS expenditure, selection of patients from waiting lists, and their management in hospitals has remained the prerogative of the individual hospital consultant, despite periodic attempts to introduce more transparent and objective selection criteria and productivity targets.

Within a year of the start of the NHS, the Ministry of Health was consciously addressing waiting lists and looking for ways to explain and reduce them. The first Circulars (government communications to NHS organisations) on waiting lists asked hospitals to review their procedures and submit reports (Ministry of Health, 1949, 1954). The Nuffield Provincial Hospitals Trust and the King Edward’s Hospital Fund for London (health service thinktanks) undertook studies on hospital efficiency − continuing a research interest that pre-dated the NHS. Some hospital boards also chose to conduct their own waiting list studies, such as in Cardiff in 1953–1954 (Grundy *et al*., 1956).

It was not until 1964 that the Ministry acknowledged an effect that economists already recognised: that an increase in consultant staffing to address the waiting lists actually stimulated increased demand (Ministry of Health, 1965). Studies of temporary policy solutions demonstrated that systems settled quickly into a new equilibrium (Aldridge, 1965; Williams, 1968). Active manipulation of waiting lists, and associated issues such as patients’ length of hospital stay, also depended upon the willing engagement of the clinician. Yet as the Deputy Chief Medical Officer, Henry Yellowlees put it: ‘There can be no telling surgeons how long their patients should be in hospital’. All that could be done was to put surgeons ‘in possession of the facts’ [Department of Health and Social Security (DHSS), 1968].

Enoch Powell, Minister of Health between 1960 and 1963, acknowledged the realities of trying to meet infinite demand, and the risks of unacknowledged rationing. On the specific issue of waiting lists, Powell noted: “I cannot but reflect sardonically on the effort I myself expended, as Minister of Health, in trying to ‘get the waiting lists down’. It is an activity about as hopeful as filling a sieve … In a medical service free at the point of consumption the waiting lists, like the poor in the Gospel, ‘are always with us’” (Powell, 1966: 39–40).

Waiting lists remained relatively steady between 435,000 and 491,000 through the 1950s and early 1960s. From 1965 to 1973 they ranged between 510,000 and 549,000. The Ministry of Health and its successor from 1968, the DHSS, conducted periodic surveys of waiting lists, occasionally uncovering evidence of hospital manipulation of statistics. The 1971 review found that not all patients who would benefit from hospital treatment had been included, and that in some hospitals patients classed as ‘urgent’ had been waiting for more than a year (DHSS, 1971).

## Waiting lists and private medicine in the NHS

The NHS, by the mid-1970s, was experiencing increasing financial pressures. The OPEC international oil crisis forced budget cuts across all public sector services, and the DHSS fought hard to preserve the *status quo* for the NHS. The election of a Labour government in 1974 reignited the debate over private medicine in the NHS, which also impinged on the waiting lists issue. After 1963 there had been no further specific Ministry/DHSS Circulars until 1975, which requested that Regional Health Authorities actively manage their lists by checking whether patients still needed surgery, and by sending data to the DHSS for national collation (DHSS, 1975). Barbara Castle (Secretary of State for Health 1974–1976), had ring-fenced £5 million of capital funding to address ‘bottlenecks’ in patient treatment in 1975–1976.

To meet their election manifesto pledge to remove private medicine from NHS hospitals, the government passed the Health Services Act in 1976 which established a Health Services Board (HSB) to oversee a reduction in ‘pay beds’. Yet, some of the demand for private medicine came from patients who wished to bypass the NHS waiting lists. As part of negotiations the government had conceded to the medical profession’s demand to abandon an end date for this process in return for their acceptance common waiting lists (both NHS and private patients).

The HSB’s report *Common waiting lists for NHS and private patients in NHS hospitals* drew attention to the ‘wide variety of waiting list systems currently in use (for example lists personal to consultants, lists common to a number of consultants in a specialty, lists for particular conditions and booking or diary systems)’. It recommended that ‘medical priority’ should be assessed according to a broader set of determinants than purely ‘clinical condition’ (HSB, 1977). It provided a list of ‘common factors’ for consideration in managing the movement of patients on waiting lists. These included:

- Pain and discomfort suffered by the patient.
- The length of time the patient has spent seeking in-patient admission.
- The patient’s domestic circumstances including his dependency on community support services (e.g. the effect his housing might have on his condition).
- The patient’s occupational circumstances (e.g. teachers seeking admission during school holidays).
- The patient’s willingness or ability to enter hospital at the time offered.
- The availability of hospital staff.
- The need to balance admissions for major and minor conditions to ensure practical waiting lists and balanced nursing workloads.
- The need to admit patients with a variety of conditions to assist professional teaching and training (particularly in teaching hospitals).

## Health economics in the NHS

There is a clear relationship between the emergence of health economics as a distinct academic discipline and profession in the United Kingdom, and rising concerns for NHS efficiency and effectiveness. Despite the lack of a classical market, the NHS provided a fertile testing ground for economic theory and from the mid-1960s a small group of economists chose to make it the focus of their research. Brian Abel-Smith was the first to apply methods of social accounting to the NHS through his role on the 1953–1956 Guillebaud inquiry (Sheard, 2013). Other economists who claimed the NHS as their fiefdom included Jack Wiseman and Alan Williams, who developed a health economics stronghold at the University of York soon after its foundation in 1963 and cultivated an influential research group, including Tony Culyer and Alan Maynard − to name two of the most prominent.

In 1976 Tony Culyer published an article with John Cullis (lecturer in economics at the University of Bath) in the *Journal of Social Policy*: ‘Some economics of hospital waiting lists’ (Culyer and Cullis, 1976). They made the first clear proposition that emphasis should be switched from the number of patients on waiting lists to the amount of time patients had to wait for treatment. Other economists had begun to explore waiting time from a theoretical perspective (Nichols *et al*., 1971; Acton, 1975). The US economist Martin Feldstein (1964), whose Oxford PhD research was on the NHS, had also attempted, with limited success, to use waiting list data as a proxy for excess demand for inpatient care. Culyer and Cullis sought to consider broader factors and to demonstrate that patients waiting for inpatient care really did pay a time price for their pain and inconvenience. They called for the development and use of a hospital admissions index to express quantitatively the patient’s need for care:

The necessary characteristics of such a concept include, first, that it should enable patients to be ranked by priority − a priority moreover, that is not lexicographic (in the sense that some ‘needs’ dominate all others and must be fulfilled first) but which allows components of the concept of need to be traded off at the margin. This immediately offers a challenge to conventional waiting-list management today, in which clinical considerations often dominate all others (Culyer and Cullis, 1976: 252)

Culyer and Cullis recognised that there were interdependencies between the supply and demand sides of the waiting list problem, especially as the doctor decided both what was to be demanded and supplied. The general practitioner (GP) was key to this process, and research by the Institute of Hospital Administrators (IHA) (1963) in 1963 had already established that their referral practices were sensitive to the length of inpatient waiting lists. Other studies had highlighted wide variations in GP referral patterns, and their considerable discretion in choosing which patients to refer and to which consultant (Forsyth and Logan, 1968; Ashford and Pearson, 1970; Hicks, 1972). At the admissions end in the hospital, in the IHA survey of 82 hospitals in England and Wales, 50 replied they had adopted agreed medical criteria for admission and 41 said no − that it was dependent on individual consultant preferences.

Culyer and Cullis’ conclusion was that:

… no generally consistent criteria for referral and admission are currently employed in the hospital service. This, in the context of the NHS, which is explicitly devoted to the efficient and fair allocation of health care resources, is an unsatisfactory state of affairs, for the case that the doctors are not the only relevant arbiters of needs (or even the most important arbiters) can easily be made in principle and it appears clearly to be the case that they are not consistent anyway in practice (1976: 255).

It was not as though decision rules for admission did not exist: model schemes, such as that by Luckman *et al*. (1969), had been developed in the late 1960s. They produced a ‘priority index’ based on how long patients had been waiting and urgency based on their expected deterioration and degree of disability. Culyer and Cullis elaborated on these ideas, stressing that selection of variables for inclusion in the index and the weights attached to them should be seen as policy decisions that embodied particular value judgements: ‘They are not matters that can be decided by social science (nor any other kind), nor are they matters, in our judgement, that should be decided by persons without public accountability’ (1976: 258). Only one of their criteria was purely clinical: urgency based upon the expected rate of deterioration of the patient’s condition. All criteria (with the exception of time spent waiting on the list which was already available from hospital records), required the use of scoring procedures. Culyer would have been familiar with a similar scoring system that his York colleague Alan Williams was then using to develop the quality adjusted life year (QALY) tool, based on the work of Rosser and Watts (1972) (Williams, 1985). The added benefit was that scoring could be done by junior medical staff, and some of the categories could even be completed by an administrator. To address concerns about maintaining case balance for teaching purposes, some flexibility in putting together theatre lists could be retained by an ‘Admitting Officer’. The index could also be used to ensure efficiency by choosing those patients who would benefit most from hospital treatment. It might result, for example, in the admission of two patients with relatively short expected lengths of stay and low index scores rather than one person with a long expected stay and a high index score.

More ambitiously, Culyer and Cullis envisioned their admissions index being used to reduce inequalities between departments, hospitals and regions. This, however, would require the establishment of national norms and ‘a substantial reduction in the discretion of individual hospital doctors’ (Culyer and Cullis, 1976: 263). They concluded that attempts to regulate waiting lists and waiting times by manipulation of indirect variables such as the bed stock, length of stay, manpower and operating theatre capacity were unlikely to be very effective. These missed the ‘crucial heart of the matter anyway, which is to ensure that admissions should be conducted so far as possible in accordance with some basic principles of efficiency and fairness’ (Culyer and Cullis, 1976: 264).

Culyer and Cullis’ (1976) paper was not a purely academic study. It was the outcome of collaboration with the DHSS. The DHSS had established its own Economic Advisers Office in 1969, and was actively developing new modes of engagement between its staff and university-based economists. Yet there appears to have been a reluctance to be transparent about the use of external expertise: Culyer and Cullis’ list of urgency criteria is almost identical that recommended in the 1976 HSB report, but their advisory role was not acknowledged in it.

The impact of the HSB report was negligible. In July 1978 the HSB issued further recommendations on moving to common waiting lists, but the medical profession again refused, complaining about lack of consultation with the Joint Consultants Committee. The Secretary of State for Health, David Ennals (1976–1979), was not in a strong enough position to force the issue (Webster, 1996: 627). In a new tack, the government set up a working party to study orthopaedic waiting lists under the chairmanship of the surgeon Robert Duthie (1981). Its report was also toothless, recommending internal district reviews. Meanwhile, waiting lists continued to rise.

During this first NHS period, 1948–1979, there was relatively little discussion of waiting lists within the medical profession, if articles and correspondence in the *British Medical Journal (BMJ)* and the *Lancet* are indicative. The *BMJ* carried just two letters in the 1950s; three in the 1960s and 12 in the 1970s. Some were written by clinicians offering practical solutions to problems they witnessed in their own hospitals, such as non-attendance, unfeasibly short call-up periods, or a failure to allow consultants to book their patients at outpatient clinics. Occasionally there is insightful dialogue between clinicians through the letters pages (Choyce, 1971; Gardiner, 1971; Harris, 1971; Fitzgerald, 1972; Harris, 1972). Rarely, there were damming critiques of hospital inefficiency, such as the *Lancet* editorial in 1976 that commented:

There are many reasons, apart from tradition, from continuing to write ‘to come again, 3 months’ in outpatient notes. Most are irrelevant to the health of the community. Few could stand up to rational examination (Lancet, 1976: 301–302; Cox, 1977).

## 1979–1997: NHS managerialism

The election in 1979 of Margaret Thatcher’s Conservative government heralded the start of a new era of central, and more politicised, interventions in the NHS. A new ‘managerialism’ was cultivated, especially following the 1983 Griffiths report which brought in general managers (Griffiths, 1983), and new analytical tools developed by ‘technicians’– economists, epidemiologists and others (Klein, 2013).

Exposure of the considerable variations in NHS practices was a key strategy of the Conservative governments. The economist C.E.B. Frost (1980b), working in collaboration with the DHSS, established that the average waiting list per consultant was around 160 patients, which represented approximately 2 months’ work. He warned that the Culyer and Cullis points-based admissions index could lead to the ‘rationally economising consultant’ who would ‘divert resources to accumulate points under the scheme as long as his value of the anticipated benefit of inpatient care exceeds the anticipated cost to him of earning the extra point …’ (Frost, 1980a: 6–7).

Frost proposed two alternatives: first, the waiting list premium − the consultant would receive payment according to the reduction in their waiting list. Second, and the more attractive option − a discharge index − which would take account of all the consultant’s clinical duties including outpatients’ appointments. Frost also supported the potentially disruptive shift in analytical focus − from waiting list numbers to waiting times:

Economists are not alone in trying to divert attention from waiting lists figures to waiting times thereby encouraging a more meaningful discussion of cost-reducing measures. The government clearly has a difficult task. It has to encourage doctors to use medical resources sparingly, and yet it has also to reassure taxpayers that their taxes have bought them medical treatment as and when they require it … If we agree that much of the concern expressed about waiting lists is really symptomatic, then possibly we can also agree that the fundamental problem is one of selecting an appropriate incentive structure for the consultants (Frost, 1980a: 7).

Studies − by health economists, and increasingly from health services management experts using Operational Research methodologies − provided evidence that governments could use to justify new policies to tackle waiting lists, both in the United Kingdom and in other countries that had waiting lists but different forms of health systems. Lindsay and Feigenbaum, for example, demonstrated that waiting on a list was a cost because a ‘good’ (i.e. treatment) received later is worth less than one received now (Lindsay and Feigenbaum, 1984; Globerman, 1991). An NHS census on 31 March 1984 revealed that there were 692,945 patients waiting to be admitted to hospital (in England), of whom 44,713 were classed as ‘urgent’. In total, 29,283 of the urgent patients had been waiting more than a month, and 194,614 non-urgent waiting more than a year. The 1975 DHSS Circular had set ‘recommendations’ for the NHS that urgent cases should be *treated* within a month, and non-urgent within a year, and that health authorities should make information on waiting lists available to GPs (DHSS, 1975).

John Yates and his Inter-Authority Comparisons Consultancy group in the Health Services Management Centre at the University of Birmingham established a spectrum of consultant surgeon performance of between 200 and 1000 operations per annum, and a variation in orthopaedic waiting lists across NHS districts from less than 100 to an extreme outlier of 4089 (Yates and Wood, 1985; Davidge *et al*., 1987). However, an inquiry by the National Audit Office found some hospitals left their operating theatres woefully underused, and up to 25% of their acute beds regularly empty (National Audit Office, 1987). The variability in performance was undoubtedly linked to the variability of NHS Districts − from small ones such as Rugby with a population of some 87,000 to neighbouring Leicester’s 860,000. These differences in need and resources exacerbated tensions between central accountability and local decision making which were increasingly exposed through regional reviews (Day and Klein, 1985).

In his book, *Why are we Waiting?* John Yates stated that “If any organisation can make a mess of collecting data it is the NHS” (1987: 9). He found evidence of hospital clerks losing patients’ appointment cards, and the national collation of data were minimal and crude. The DHSS deposited its collations in the House of Commons library, and considered that sufficient for ‘publication’. In 1985 the long-running Hospital Inpatient Enquiry, which generated the inpatient statistics, was radically reformed. Yet no data had ever been collected on waits for outpatient department appointments, and inpatient waiting lists excluded day surgery patients and those with booked appointments.

## The College of Health Guide to Waiting Lists

A response to the rising waiting lists, and their variation within regions and by surgical specialties came from the College of Health, which Michael Young founded in 1983. It was a non-profit-making body intended to complement the Royal medical colleges and give those on the receiving end of health care a source of support (it published a regular magazine ‘Self Health’). The first edition of *The Guide to Waiting Lists* was issued by the college in May 1984, and was updated annually until 1991 (College of Health, 1984).

In part, the *Guide* was created in response to a British Medical Association (1984) survey of all NHS districts in England and Wales between April 1983 and April 1984 that had found patients were waiting up to 3 years simply to get an outpatients appointment.^3^ There was increasing concern that patients were seeking private treatment to jump the lengthening NHS queues. Although the numbers were not large − 81,000 private patients were treated in the NHS in 1982 − it created ethical dilemmas, especially for hospital consultants (College of Health, 1984).

The *Guide* collated waiting time statistics (percentage waiting more than 1 year) from each NHS district by the eight main surgical specialties. These were arranged in comparative tables, showing districts with 20% shortest and longest waits, enabling patients and their GPS to see if they would get faster treatment through a referral to another NHS district, or even region. It also provided a list of questions for the patient to ask the consultant to establish whether a private referral would be of benefit. The College of Health voiced concerns that increasing pressure on the NHS would stop the prestigious London teaching hospitals accepting patient referrals from around the country. The public were not routinely provided with information on their rights of referral, or even advised that their travel costs could be reimbursed. It concluded that ‘Best of all would be a computerised data bank, with terminals in every GP’s surgery so that they could see just what the current situation was in many hospitals before deciding which one to refer a patient to’ (College of Health, 1984: 26).

The Department of Health (DH; successor to the DHSS in 1988) did later provide funding for a pilot study on computerised waiting list information system for GPs and patients. However, it discontinued funding after the first year. It maintained a policy of targeting waiting lists by short-term injections of financial resources. Beginning in 1985, the Waiting List Initiative spent £252 million until its termination in 1995. The Secretary of State for Health, Norman Fowler (1983–1987), authorised an additional £30 million investment between September 1987 and March 1988 to reduce the list by 100,000. However, the list actually grew over that period from 690,000 to 704,000, an increase of 1.9% (Klein, 2013). In 1990, 100 new consultant posts were created, ignoring strong evidence from health economists that this would lead to further supplier-induced demand.

## 1991–1997: impact of the NHS internal market

The NHS underwent major reform enabled by the 1990 National Health Service and Community Care Act which created the ‘Internal Market’ of purchasers and providers. Performance indicators, introduced in the 1980s, became significant drivers in improving NHS efficiency. In 1991 a Patient’s Charter established a right to treatment within 2 years of diagnosis: a significant commitment to shift the target from number of patients on waiting lists to waiting *times* (Secretary of State for Health, 1991). As with other policy initiatives, it was developed in an ‘evidence vacuum’ with no acknowledgement of what was by this stage a considerable amount of research, especially from the health economics community, on how waiting lists developed. The NHS Management Executive from 1991 sent out annual guidance on priorities, requiring health authorities to deliver reductions in waiting times. Wide variations in GP practices were still common: rates of referral ranged from 2.5 to 5.4 per 100 consultations and from 4.3 to 13.2 per 100 patients on GP lists. GP fundholding did little to change referral patterns (Farrow and Jewell, 1993: 67; Surrender *et al*., 1995). Access to hospital care seemed to be ‘largely a function of how individual GPs interpret their gatekeeping role’ (Klein *et al*., 1996: 85). Some of this was due to GP competence and confidence. John Fry (1978) noted that he reduced his referral rate from 105 to 47 per 1000 patients over 25 years, and attributed this to his improving diagnostic competence, as well as knowledge of hospital consultant capabilities.

In a new policy tack, the DH began to publish comparative hospital data in the form of an annual guide that gave star ratings for individual hospitals. One of the criteria for rating was the size of the waiting list (NHS Executive, 1994). The DH also initiated routine use of clinical audit to demonstrate clinical effectiveness, but this drive for efficiency depended on the cooperation of the medical profession (Kerrison *et al*., 1994: 185; Day *et al*., 1998).^4^ The 1991 reforms reinforced the continued dependence of the government and NHS managers on clinicians, rather than permitting a significant shift in authority (Aaron and Schwarz, 1984). Ministers and managers could “shelter behind the doctrine of clinical judgement … doctors in turn internalised scarcity in their judgements about appropriateness [of treatment]” (Klein, 2013: 176).

A more interventionist policy for waiting lists was sanctioned by the Secretary of State for Health, William Waldegrave (1991–1995), who commissioned John Yates’ Inter-Authority Comparisons Consultancy to work with the 22 worst performing NHS districts. Their micro-management project cost £9 million, which was subsequently calculated as a price of £1 million per 2000 reduction in list size. The government quietly abandoned the initiative (BMJ, 1991; Yates, 1991; *BMJ*, 1992). Under such intense scrutiny, some districts adopted devious tactics: North West Thames was found to have abolished its varicose veins waiting list; some districts made patients wait longer for outpatient appointments to try to ‘stop the clock’, or temporarily suspended their admission (Pope, 1992).

Academic research demonstrated that supply side factors were also significant, and highlighted that hospital consultants were still managing their lists using obscure priorities (Propper, 1990; Iverson, 1993, 1997). Although most hospitals operated a three point scale of urgency − immediate, urgent and non-urgent − there was no clear understanding of how consultants interpreted these definitions (Evans, 1990; Harvey, 1993). Stephen Frankel and Robert West claimed that this opaqueness over waiting lists permitted “a blurring of the National Health Service’s capacity for or commitment to certain sorts of treatment. Waiting lists veil the discrepancies between what is offered and what can be done”. They compared waiting lists to a mortlake formed by a fast flowing river, in which patients with ‘uninteresting’ surgical needs such as varicose veins and hernias languished (Frankel and West, 1993: 44–62). A study of consultant prioritisation practices undertaken by Alistair Lack and Sarajane Fletcher (Lack & Fletcher, 1995) at Salisbury hospital found that the most important factor considered was the progression of the disease, alongside lesser factors such as degree of pain and disability and the effect on the patient’s capacity to function.

## 1997: New Labour

The ‘New’ Labour government under Tony Blair from 1997 introduced a much tougher approach to the NHS through the use of formal performance management. As Rudolf Klein neatly puts it: ‘If priorities were the language of Socialism (according to Aneurin Bevan), targets were the language of New Labour’ (Klein, 2013: 201). The Labour party had prioritised NHS efficiency in their election manifesto, making a specific promise to cut the waiting list of 1,160,000 by 100,000 in their first year in office. In total, £500 million was made available to tackle related issues, through greater use of the private sector and financial incentives for hospitals in a ‘payments by results’ strategy. However, by March 1998 the waiting list had grown to 1,297,000 and the Secretary of State for Health, Frank Dobson (1997–1999), found himself the butt of political and media jokes. Although there was subsequently a recorded improvement, it later emerged that this had been manipulated by increasing the (unmonitored) time patients waited before their first hospital appointment. The National Audit Office found that waiting list statistics had been ‘inappropriately adjusted’ in nine trusts (Comptroller and Auditor-General, 2001). A follow up spot check of 41 trusts by the Audit Commission (2003) found evidence of deliberate mis-reporting at three trusts and reporting errors in 30% of all waiting list performance indicators examined.

In 1998 a study by the British Medical Association’s Health Policy and Economic Research Unit (1998) advocated for the adoption of a priority scoring system such as that used in New Zealand (from 1996) and Sweden (Hadorn and Holmes, 1997; Fricker, 1999). This was supported by research done over the previous two decades by health economists and management experts (Feldman, 1994). It was noted that UK local authorities had long used priority scoring effectively for allocating public sector housing (Edwards, 1997). And there were some clinicians willing to undertake pilot studies in collaboration with economists. At Guy’s hospital in London Claire Gudex and Alan Williams applied a scoring system for patients’ expected net QALY and used it to rank surgical conditions (Gudex *et al*., 1990; Lack and Smith, 1995; Lack *et al*., 2000). Although there were difficulties with clustering of conditions for preparing operating theatre lists, a study at Carmarthen hospital showed that this could be overcome by introducing a patient initial quotient to determine whether a patient should be placed on a list and an algorithm to reflect time waited which led to a more balanced case mix. A modelling exercise at Salisbury hospital showed that applying a ‘Points Scheme’ to a ‘first come first served’ orthopaedic waiting list produced considerable changes in the order of patients to be treated: only seven patients appeared in the first 20 patients selected under both schemes. The Salisbury scheme required fewer resources to treat its first 20 patients, but eliminated fewer days of waiting from the list (Edwards, 1999). These studies highlighted the need to consider wider philosophical and ethical issues, including whether clinicians would ‘game’ a priority points system for their patients’ benefit. As the health economist Rhiannon Tudor Edwards noted about priority scoring systems in 1999:

Nevertheless, before their widespread introduction we need to evaluate their dynamic effects over time on case mix, distribution of waiting times, and patterns of resource use. This will involve looking within the ‘black box’ of NHS waiting list management to find out far more about the beliefs and behaviour of those involved in the delivery and receipt of elective health care in the NHS (Edwards, 1999: 413).

## 2000: from waiting lists to waiting times

In 2000 the Chancellor of the Exchequer, Gordon Brown announced a ‘step change in resources’ to coincide with the publication of the NHS plan. This included a radical shift from monitoring by numbers on waiting lists to lengths of waiting times. An ambitious target was set to reduce maximum waiting time (from placement on inpatient list) from around the current 18 months to 6 months by the end of 2005 (and 13 weeks for an outpatient appointment). This was a phased target, with annual goals: 15 months by 2003; 12 months by 2003 and 9 months by 2004. Alan Milburn, Secretary of State for Health (1999–2003), declared a ‘war on waiting’. His understanding of the complexities of NHS waiting appeared limited, judging from his use of supermarket queuing analogies, but it did not detract from a very aggressive campaign focussed on NHS staff, especially hospital managers, who were subjected to harassing phone calls from DH as a part of a concerted ‘Targets and Terror’ strategy for punishing those who failed to meet waiting time targets. This was likened to the targets set for managers of state enterprises in the USSR (Bevan and Hood, 2006; Propper *et al*., 2008).

Yet although waiting times did begin to fall, research showed little actual increase in overall hospital activity: targets were being met by distorting clinical priorities − taking the longest waiters in advance of those with more urgent clinical needs (Harrison and New, 2000). The government had also come to a concordat with the private sector and used Independent Sector Treatment Centres for block contracts of hip replacements, and other relatively uncomplicated surgical procedures. Private medicine was alive and well in NHS hospitals, and now actively encouraged by New Labour: it brought in revenue for the NHS and provided an incentive for some consultants to stay within the system.

Health economists responded to this significant policy shift from waiting lists to times by critiquing the choice of analysis the DH was performing internally and/or commissioning (often from their academic units). To them, it was not clear that the right questions were being asked of a health care system that appeared to be in a chronic state of financial breakdown: there was little focus on bigger issues such as how much to spend on what sorts of treatments, and who should have priority in what was clearly a rationed (by waiting) system. Intermittent international comparative studies highlighted the opportunities offered by robust and transparent priority setting systems, such as that used in New Zealand (Cullis *et al*., 2000; Devlin *et al*., 2002a, 2002b; Martin *et al*., 2003; Appleby *et al*., 2005).

Politicians appeared to prefer tinkering through relatively minor adjustments to the monitoring of waiting, rather than attempting to introduce new schemes to achieve efficiency through prioritisation. John Reid (Secretary of State 2003–2005) changed the statistical goalposts by requiring hospitals to report on Referral to Treatment Time (RTT), which covered the whole ‘patient pathway’ from GP referral, rather than from when a hospital consultant placed a patient on a list for admission to hospital. The NHS Modernisation Agency (founded in 2000) worked with hospital trusts to use new tools such as Patient Tracking Lists. A new RTT target of 18 weeks was set. Reporting was now to include *all* elective care, including booked cases. More ambitious targets were set for high priority services such as cancer and heart disease. The statistics published following the introduction of these new targets showed clear quarterly peaks in admissions, coinciding with dates of hospital reporting to DH. Admissions managers practiced ‘gaming’ by temporarily suspending patients from lists, and reinserting them after the reporting dates.

Meticulous, sustained analyses by health economists such as Carol Propper *et al*. (2008) demonstrated how within the new internal market culture of the NHS, pressure to meet waiting targets (which were measured and thus a factor in fundholder referrals) were prioritised, and which resulted in declines in unmeasured quality (as expressed in their study of acute myocardial infarction mortality). Another component of the internal market − the option for patients to choose their place of treatment (fully operational from 2005) − could have helped to level out the extreme of waiting times between NHS authorities. Yet early evaluation found that the majority of patients preferred to use their local hospital, and were prepared to sacrifice quicker access for longer waits at hospitals they were familiar with because of proximity or reputation (Taylor *et al*., 2004; Burge *et al*., 2005; Lewis and Appleby, 2008).

In 2009 the last significant target of a maximum 18 week GP referral to treatment in hospital was achieved. Since then, the pattern has remained one of intermittent short-term firefighting to keep within target *time* range, but the numbers of patients waiting has increased, and now stands at some 3.4 million (Campbell, 2017). And on 31 March 2017, the Chief Executive of the NHS for England, Simon Stevens, announced that the 18 week target for some surgical procedures (hip or knee replacement, cataract removal, hernia repair and other non-urgent conditions) would have to be ‘relaxed’ to divert resources to parts of the health service with greater need (mental health, GP access, cancer services).

## ‘Space’: waiting as an international comparative analysis tool

The history of waiting policies can also illuminate the development of the international comparative analysis in health care. Although policy theorists such as Theodore Marmor *et al*. (2005) have been sceptical of the genuineness of much scholarly activity that claims to be comparative analysis, the salient point is that such activities have not shown a linear progression. Although there were early pioneers, such as Brian Abel-Smith who began consultancy work for the World Health Organisation in 1956 (producing a comparative study of health care expenditure in six countries), there were relatively few adopters until the 1990s (Sheard, 2013). The history of international comparisons, and why they seemed more attractive then, rather than during the fiscal crises of the mid-1970s and mid-1980s, deserves further analysis.

Waiting for inpatient treatment did not receive systematic international comparative analysis until the Waiting Times Project conducted by Luigi Siciliani and Jeremy Hurst at the Health Policy Unit of OECD in 2001–2003 (Hurst was on secondment from the UK DH) (Siciliani and Hurst, 2003, 2005). Their 12-country study demonstrated that both supply and demand policies could reduce waiting times, but could also provoke tension between clinicians and policymakers. They also found preliminary evidence that increased private health insurance coverage reduced waiting times. National waiting lists problems also allowed health economists to broaden their international horizons, as evidenced by citations of foreign studies in their academic papers and consultancy briefs for government departments. For waiting policy in the United Kingdom, the gaze frequently turned to Canada, Scandinavian countries and New Zealand, and the gaze was returned from these countries (Appleby *et al*., 2005; Willcox *et al*., 2007).

## Waiting for inpatient treatment in Canada

This brief overview of one of these regular ‘comparator’ countries − Canada − puts the UK waiting history into perspective. Although the adoption of a Medicare system in 1960s established a form of universal health care coverage, there was scope for considerable variation at the provincial level, and the issues of lengthening waiting lists and geographical equity emerged as a significant policy and political issue in the 1980s and stimulated a range of solutions. For example, the waiting list crisis for coronary artery bypass surgery in Ontario led to the creation of the Cardiac Care Network (Naylor, 1991). In British Columbia a similar problem led to a contractual arrangement between the Ministry of Health and four Seattle (US) hospitals to provide surgery for up to 200 cardiac patients a year (Katz *et al*., 1991). Alberta and Quebec also had lengthy queues for cardiac surgery and paediatric services. Some very personal accounts made media headlines and heightened pressure on politicians to take action, as in 1990 when several patients in British Columbia died while on a waiting list for heart surgery (DeCoster *et al*., 1999).

Waiting lists have often been portrayed in Canada, as in the United Kingdom, as a symptom of inadequate funding. Provincial governments found to their cost that reducing resources for diagnostic tests led to lengthening wait times for elective surgery. There were increasing concerns about overcrowding in Emergency Departments and in other parts of the Medicare system. Despite *ad hoc* increased in resources, often targeted at specific procedures such as hip replacements, ongoing uncertainties underpinned calls for radical reforms of how health care is delivered, such as those issued by the Frazer Institute. Yet detailed studies demonstrated that this was not always a system wide, or national issue. In Nova Scotia, for example, in the late 1990s, waits for some services including hip and knee replacements had in fact fallen (Nova Scotia, 1997). This uncertainty over the accuracy of waiting list data led to Health Canada commissioning a report in 1998 to review and synthesise information. It found widespread interest in and support for standardising data and coordinating waiting lists, and that significant investment was needed in waiting list infrastructure (McDonald *et al*., 1998).

The Western Canada Waiting List Project, which reported in 2001, drew on international expertise, including John Yates from the United Kingdom and David Hadorn from New Zealand. In total, 19 partner organisations collaborated to develop scoring tools for admissions priority setting. Significantly, members of the public were involved in this process, helping to establish concepts of need and importantly, fairness in access to health care. Many of the public participants had no idea that there was no national system of waiting list management, or collection of data on waiting (Western Canada Waiting List Project, 2001; Noseworthy *et al*., 2003). In 2002 the Canadian government invested $5.5billion to decrease waiting times as part of a 10-year strategy. In 2005 health ministers produced six waiting time benchmarks for specific procedures that had been identified in the 2004 10-year plan (Marchildon, 2013). In 2007 Prime Minister Stephen Harper announced that all provinces and territories would establish patient waiting time guarantees by 2010 for up to five key treatments: cancer care, hip and knee replacement, cardiac care, diagnostic imaging, cataract surgery and primary care.

Health care in Canada has also struggled with issues of gatekeeping and the role and impact of private medicine. A study of waiting times for cataract surgery in Manitoba demonstrated that waiting lists were longer in districts that permitted surgeons to practice in both public and private arenas, but it also found that the public had little accurate understanding about trends in waiting, and were often misinformed by thinktanks such as the Fraser Institute which were supportive of a move towards a more marketised health care system (DeCoster *et al.*, 1999). In 2013 the Vertes Commission judicial enquiry into queue jumping in Alberta provided a rare platform for discussing how routine processes and structures in Canadian health care lead to preferential and differential access. Vertes recommended that the public system needed to standardise and strengthen wait list management strategies, and referral triage and booking systems, including access via the emergency departments to specialist care (the so-called ‘private patient path’); better definition of queue jumping and protection for whistle blowers (Reid, 2017).

As in the UK NHS, piecemeal initiatives have not achieved a significant, sustained reduction in waiting times. According to the 2016 Commonwealth Fund international survey of 11 high-income countries, Canadians have the longest wait for primary and specialist care, with 30% waiting more than 4 weeks to see a specialist (Osborn *et al*., 2016).

## Waiting lists, times and policy analysis frameworks

Three key policy lessons emerge from the history of waiting lists and times in the United Kingdom. First, a failure to acknowledge and address tensions between the different NHS policy levels (intra-/inter-regional and national) will affect the relative success of performance management techniques. Second, radical policy changes should be piloted, even if this is uncomfortable/risky for policymakers. Third, conscious effort needs to be invested by policymakers in ensuring all expert groups continue to be actively engaged in policy formation.

Culyer and Cullis made the first serious proposal to meet expressed need for UK NHS services by a comprehensive and transparent priority scoring system for admission from waiting lists in 1976. They observed wide variations in NHS performance at all levels: regions, districts, hospitals and individual clinicians. More than 40 years later, there is still no ‘NHS-wide’ admissions system.

In the intervening period there have been several significant policy initiatives: intermittent injections of funding, use of targets and private sector capacity, shift from focus on waiting list size to waiting time. There have been parallel, but not always related, research initiatives, the majority led by health economists, to distinguish demand and supply factors, but these have often been hampered by poor data, or skewed by the personal academic research interests of those undertaking the projects (and the implicit analytical framework has been Paretian − focussed on welfare economics). Economists have been critical of policymakers for failing to establish an explicit set of criteria against which to evaluate policy options, for their failure to recognise the problems created by using targets (feedback effects, perverse incentives) and their failure to be clear about whether the policy goal is health service efficiency or equity (Cullis *et al*., 2000; Devlin *et al*., 2002a, 2002b; Lewis and Appleby, 2008). They have been increasingly critical of the medical profession, especially those clinicians who have resisted the introduction of priority scoring indices. But these critiques ignore the subliminal, and usually messy, nature of policy development. Few health economists have acknowledged one of the main weaknesses of the UK (and NHS) policy culture, which is to bypass pilot studies and to go for what Carolyn Tuohy calls the ‘big bangs’: making whole system changes, as occurred with the introduction of the internal market in 1991 (Tuohy, 2017a).

Discussion of, and responses to, waiting lists and waiting times has varied since 1948. There is a correlation not only between increasing waiting and amount of discussion, but also with the improvement and accessibility of statistics. In the early years of the NHS the scale of the problem was reported annually, and it was not easy to ascertain the extent of variation within the NHS. Local waiting list issues, as in Cardiff in 1954, initially triggered local responses. It was not until a single, ‘national’ waiting list figure was produced that national policy solutions were considered and/or attempted. Even then, there have been issues with what is deemed relevant to collect statistics for: waiting for inpatient treatment has been only part of the picture (and did not include those patients booked at time of decision to treat), but it was not until 1986 that information on waiting for outpatient appointments was collected, and 2005 that policies specifically addressed ‘Referral to Treatment’, or the whole ‘patient pathway’ were introduced.

Reliability − or rather malleability − of statistics for waiting lists and times appears to have been responsive to political pressures. Hospitals were found to be mis-reporting their performance from the 1960s onwards. Even in the 2000s there were problems with evaluating hospital level performance, amid concerns of clinicians and managers ‘gaming’ the system, and government reluctance to define the ‘elective care’ they were measuring (Harrison and Appleby, 2009; Findlay, 2017). Clinician-level performance data poses another set of ideological challenges. Relativity has also been an important factor: both within and between the NHS regions and, since the 1999 devolution of health care, between the four UK nations. This opened up new analytical possibilities for health economists and other researchers, who were now able to compare, for example, the use of targets (England) with no targets/or rankings (Scotland and Wales) (Hauck and Street, 2007; Propper *et al*., 2008; Bevan, 2010).

Addressing both national and local waiting list and waiting time issues has been a chronic source of tension in the UK NHS. Repeated local studies demonstrated that it was difficult to attribute blame to single factors, or to clear disparities between supply and demand. At a national level, the history of waiting lists and times helps to illuminate key policy issues. First, the spectrum of ministerial determination and capacity for policy change: from Enoch Powell’s recognition of the futility of effort in the 1960s; Barbara Castle’s bravado in 1974 and 2 years later David Ennal’s timid climb-down in the face of robust medical profession defence of their clinical autonomy. In the 1980s and 1990s fluctuations in waiting lists and times became sticks with which opposition parties could beat the incumbent Secretary of State for Health (especially Frank Dobson in 1998); others resisted setting such clear national targets, and put the spotlight on local implementation. See, for example, Virginia Bottomley’s comment in 1994 that ‘we only set the framework …’ (Klein *et al*., 1996: 131).

In policy analysis frameworks, these historical fluctuations in political strength align well with John Kingdon’s (1995) Multiple Streams Analysis. They also resonate with Carolyn Tuohy’s (1999, 2017a, 2017b) evolving analysis, in which she identifies four types of policy change: big bang, blueprints, mosaics and incremental. These reflect the magnitude of change: its scale and pace, as well as how political actors individually and collectively assess their current and future political influence. Tuohy’s resultant ‘strategic domains’ enhance Kingdon’s approach by identifying not just when the ‘policy windows’ open, but when they are used (and not used). In the history of waiting lists and times, there have been few clear ‘windows of opportunity’ when government ministers had the option to impose radical change on the medical profession, such as a national priority scoring system for hospital admissions. They had the research evidence this was effective from 1976, but continued to pursue an ‘incremental’ strategy, hoping clinicians would adopt good practice through choice. This could also be seen as evidence to support David Wilsford’s (1994) concept of ‘path dependency’: that it becomes progressively harder to achieve significant policy change as tradition embeds practices. The ongoing significant variations in clinical output and waiting times did not appear to be seen by politicians as sufficiently strong justification to trigger radical policy shifts (perhaps because these had been adopted into the NHS on its formation in 1948). The policy choice was to use waiting list/time targets instead (and rankings in Scotland and later in Wales), which were focussed on hospital managers rather than to attempt direct engagement with the medical profession.

Here, Paul Sabatier’s (1988) Advocacy Coalition theory also has explanatory potential. Government ministers increasingly looked to form alliances with alternative sources of policy expertise. Their engagement with health economists was ‘incremental’, in Tuohy’s language, from the 1960s, through isolated research commissions, until the formation of DHSS Economic Advisers’ Office in 1969 and centres such as the University of York Centre for Health Economics (founded in 1983) which, acting together as commissioner and consultancy service, provided a series of potentially influential research reports that linked waiting lists and times with evidence of weak and inequitable clinical practices, especially on admissions systems. This ‘advocacy coalition’ suited both parties: government ministers benefited from a new authoritative voice which justified rationing access to the NHS by time; the health economics community benefited from an increased sense of professional identity and security. As this coalition matured, it permitted a subtle (and sometimes not so subtle) distancing of the government from the medical profession. Yet the strength of the politician/health economist coalition never seriously challenged the authority of the individual clinician/patient coalition, which trumped any national imposition of standardised management of waiting lists.

Waiting list and waiting time policies are some of the earliest forms of target setting within health services: implicit until the 1980s, and then progressively exposed through the New Public Management reforms and, in the UK NHS, the use of Performance Indicators (Bevan and Hood, 2006; Hood, 2007). They have had clear local and national dimensions: often set centrally, but negotiated at regional, district, hospital and even individual clinician levels. As targets, they can help to expose synergies and tensions between ‘governance logics’. Hierarchist governance − in which targets are imposed centrally − is often seen as mutually exclusive to experimentalist governance, in which target setting can be used as an active learning process, and the outcome of negotiations between central regulators and local authorities (Wismar *et al*., 2008; Bevan, 2010; Schang and Morton, 2017).

Historical analysis of waiting lists and times − especially of sporadic management initiatives such as increases in system capacity and use of ‘discretionary’ policies such as priority scoring indices for admissions − illuminates the relative authorities resting at the local and national governance levels: the clinician, the hospital manager, the regional director and ultimately the government minister. It highlights how the dialogue between and within these groups has changed: from concerns about ‘productivity’ to ‘need’ and ‘fairness’. While crude increases in numbers of patients/time waiting for inpatient elective treatment can be taken as evidence of under-resourcing; the extensive variations in waiting lists and times, in referral practices and in clinician/hospital efficiency expose the ‘sub-national’ reality of the NHS in the United Kingdom. Nuanced historical analyses − which lay bare the real dilemmas and battles of developing and implementing health policy − offer a valuable analytical tool for contemporary policymakers.

## Footnotes

1 Although it is common to talk about the British NHS, in reality there have been separate systems for England and Wales, Scotland and Northern Ireland. In 1999 Welsh devolution created a separate NHS Wales, so there are now four health services, with limited ‘UK’ co-ordination through Whitehall.

2 Waiting ‘lists’ traditionally contained the names of those people whom specialists had decided required in-patient treatment. Waiting ‘times’ can mean the length of time a patient has waited for admission after the specialist has made the decision to treat them as an inpatient, but more recently in the United Kingdom it has included the time waited after the general practitioner has referred the patient for a specialist’s opinion. Waiting for other types of health care, such as in Emergency Departments or for general practitioner appointments, is beyond the scope of this paper.

3 The information for Scotland and Northern Ireland was not comparable with England and Wales − it was collected at different intervals and in different formats.

4 The medical profession’s self-regulation of audit was a sign of some easing of pressure during Stephen Dorrell’s tenure as Secretary of State for Health (1995–1996).
